# Potential Effects of Natural H_2_S-Donors in Hypertension Management

**DOI:** 10.3390/biom12040581

**Published:** 2022-04-14

**Authors:** Eugenia Piragine, Valentina Citi, Kim Lawson, Vincenzo Calderone, Alma Martelli

**Affiliations:** 1 Department of Pharmacy, University of Pisa, 56126 Pisa, Italy; eugenia.piragine@farm.unipi.it (E.P.); valentina.citi@unipi.it (V.C.); vincenzo.calderone@unipi.it (V.C.); 2 Biomolecular Sciences Research Centre, Sheffield Hallam University, Sheffield S1 1WB, UK; k.lawson@shu.ac.uk; 3 Interdepartmental Research Centre “Nutraceuticals and Food for Health (NUTRAFOOD)”, University of Pisa, 56126 Pisa, Italy; 4 Interdepartmental Research Centre of Ageing, Biology and Pathology, University of Pisa, 56126 Pisa, Italy

**Keywords:** hydrogen sulfide, polysulfides, isothiocyanates, garlic, *Alliaceae*, *Brassicaceae*, hypertension

## Abstract

After the discovery of hydrogen sulfide (H_2_S) in the central nervous system by Abe and Kimura in 1996, the physiopathological role of H_2_S has been widely investigated in several systems such as the cardiovascular. In particular, H_2_S plays a pivotal role in the control of vascular tone, exhibiting mechanisms of action able to induce vasodilation: for instance, activation of potassium channels (KATP and Kv7) and inhibition of 5-phosphodiesterase (5-PDE). These findings paved the way for the research of natural and synthetic exogenous H_2_S-donors (i.e., molecules able to release H_2_S) in order to have new tools for the management of hypertension. In this scenario, some natural molecules derived from *Alliaceae* (i.e., garlic) and *Brassicaceae* (i.e., rocket or broccoli) botanical families show the profile of slow H_2_S-donors able to mimic the endogenous production of this gasotransmitter and therefore can be viewed as interesting potential tools for management of hypertension or pre-hypertension. In this article, the preclinical and clinical impacts of these natural H_2_S-donors on hypertension and vascular integrity have been reviewed in order to give a complete panorama of their potential use for the management of hypertension and related vascular diseases.

## 1. Introduction

The discovery of hydrogen sulfide (H_2_S) as an endogenous gasotransmitter, by Abe and Kimura in 1996, represents the milestone for a novel field of research which had a great impact on physiopharmacology [1]. The last 25 years have identified H_2_S as fundamental for the homeostasis of several systems, but one of the most important areas in which the role of H_2_S has been investigated is the cardiovascular (CV) system [2,3,4,5,6,7]. Indeed, since its discovery, H_2_S was considered a “relative” and a “deputy” of the best known gasotransmitter nitric oxide (NO), which, in the 1980s, revolutionized cardiovascular physiology and pharmacology [8]. On the bases of this analogy, the cardiovascular effects of H_2_S became a hot topic. This interest led to many studies focusing on clarifying the similarities, the differences, and the eventual cross-talk between H_2_S and NO at the cardiovascular level [9,10,11,12,13]. In particular, the findings that have emerged from the last two decades of investigation led to the discovery of a pivotal role for H_2_S in the control of the vascular tone. H_2_S exhibited the ability to induce vasodilation due to the involvement of mechanisms of action such as the activation of potassium channels, e.g., Kv7 or KATP, and by inhibiting 5-phosphodiesterase (5-PDE) enzymes [14,15,16]. More recently, another mechanism of action accounting for the vasodilating effect of H_2_S has been explored, leading to the discovery of an interesting role of the vascular endothelial growth factor receptor 2 (VEGFR2) [17,18]. On the basis of these vasodilating mechanisms of action, an anti-hypertensive role for H_2_S has been investigated, finding that hypertension may be due, at least in part, to a deficit of endogenous H_2_S [19,20]. This observation paved the way to research of natural and synthetic exogenous H_2_S-donors (i.e., molecules able to release H_2_S) in order to have new tools for the management of hypertension [21]. This led to the discovery that some natural molecules derived from *Alliaceae* (i.e., garlic) and *Brassicaceae* (i.e., rocket or broccoli) botanical families show the profile of slow H_2_S-donors (i.e., suggesting that they may exhibit a H_2_S-releasing profile more similar to that of the gradual endogenous production of this gasotransmitter). This feature suggests that the herbal extracts or the purified molecules (polysulfides or isothiocyanates) derived from *Alliaceae* and *Brassicaceae* could be interesting tools for management of hypertension or pre-hypertension. In this review article, the preclinical and clinical impacts of these natural H_2_S-donors on hypertension and vascular integrity have been reviewed in order to give a complete overview of their potential use for the management of hypertension and related vascular diseases.

### Mechanisms of Action Accounting for the Anti-Hypertensive Role of H_2_S

**Figure 1:**
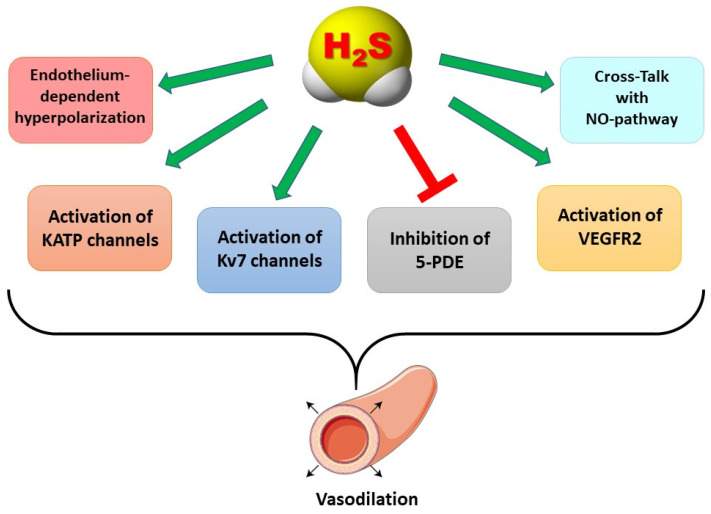
Relevant mechanisms of action accounting for the vasodilating effect induced by H_2_S. KATP = ATP-sensitive potassium channels, Kv7 = voltage-gated potassium channels, 5-PDE = 5-phosphodiesterase enzyme, VEGFR2 = vascular endothelial growth factor receptor 2, NO = nitric oxide.

H_2_S exhibits the chemical features of a reducing agent which are directly related to antioxidant properties and, in addition, has been hypothesized to induce most of its effects at a vascular level by *S*-persulfidation (often reported as *S*-sulfhydration, even if this form is not correct) of proteins. Among these proteins are ion channels, enzymes, and receptors which, as a consequence of the H_2_S-induced *S*-persulfidation, undergo the conformational change responsible for their activation or inhibition [22]. During the years immediately following the discovery of H_2_S as an endogenous mediator, potassium channels were among the targets most investigated as cardiovascular modulators [23,24,25]. One of the first mechanisms of action accounting for the vasorelaxing effect induced by endogenous H_2_S and exogenous H_2_S-donors used as experimental tools (i.e., the salt NaHS), was the activation of ATP-sensitive potassium channels (KATP) [14]. The KATP channel was probably the most investigated subtype because of the availability of well-known KATP-blockers such as the sulfonylurea, glibenclamide, mainly used as oral hypoglycemic drug, and of several series of newly synthesized chemical KATP-openers [24,26,27]. In the first study of H_2_S and the activation of KATP channels, the authors described a reverse journey from the in vivo demonstration that H_2_S-induced blood pressure lowering (inhibited by glibenclamide), the in vitro experimental models represented by dilation of rat aortic tissue, hyperpolarization of isolated vascular smooth muscle cells and expression of cystathionine γ-lyase (CSE, one of the most important H_2_S-generating enzymes at the cardiovascular level) in vascular smooth muscle cells [14]. After this first demonstration, other studies focused their attention on the interaction between H_2_S and KATP channels confirming their involvement in the induction of vascular smooth muscle hyperpolarization recorded by electrophysiological measurements [28] and exploring this mechanism of action on distinct vessels such as cerebral arterioles or the hepatic artery [29,30]. As H_2_S acts by inducing *S*-persulfidation of proteins, it is quite unlikely to lead to selective activation of just one subtype of potassium channel. A study on perivascular adipose tissue demonstrated that this tissue releases an adipocyte-derived relaxing factor (ADRF), suggested to be H_2_S, which can open voltage-gated potassium channels in peripheral arteries [31]. On the basis of this hypothesis, other potassium channels have been evaluated as potential targets for the vascular effect of H_2_S. This investigation resulted in the discovery of the activation of voltage-gated potassium channels belonging to the family Kv7 as a further important mechanism of action accounting for the vasodilatory effects. Fluorometric studies on human aortic smooth muscle cells (HASMC) and techniques carried out on rat aortic rings recording Rb^+^ efflux (as mimetic for K^+^ efflux) after H_2_S-donors administration confirmed the involvement of Kv7 channel activation in the vasodilation induced by H_2_S. Moreover, electrophysiological experiments suggested that among the different subtypes within the Kv7 channel family the activation of Kv7.4 could represent a key mechanism in the vascular effects of H_2_S [15,32]. After this first demonstration, the interaction between H_2_S and Kv7 channels was investigated in several studies focused on the pharmacological demonstration of the H_2_S-donor properties of novel synthesized or plant-derived molecules. In particular, the vascular characterization (from HASMC to isolated vascular beds such as rat aorta or coronary arteries) of the H_2_S-mediated effects of isothiocyanates or thioureas resulted in the identification of the Kv7 channel activation as the main mechanism accounting for vasodilation [33,34]. Furthermore, the activation of Kv7.4 by H_2_S, and the consequent vasodilation has been suggested as the mechanism used by the porcine coronary artery to counteract experimentally induced hypoxia [35]. The activation of Kv7 channels by H_2_S-donors such as the morpholine GYY4137 and the salt Na_2_S, which are well recognized H_2_S-donors used as experimental tools, was demonstrated in rat small mesenteric arteries [36]. *S*-persulfidation also seems to be the mechanism behind the inhibition of 5-PDE. Indeed, the ability of H_2_S to inhibit 5-PDE was first demonstrated by Bucci and colleagues on rat aortic rings by recording the cGMP levels evoked by endogenous H_2_S (or H_2_S-donors) in the absence or in the presence of specific inhibitors of H_2_S-biosynthesis such as dl-propargylglycine (PAG) [16]. Subsequently, Sun and colleagues demonstrated that the vasorelaxing effect observed on rat aortic rings after inhibition of 5PDE was due to the sulfhydration-associated PDE 5A dimerization [37]. Moreover, H_2_S has also been described as an endothelium-derived hyperpolarizing factor (EDHF) because it was observed that exogenous H_2_S hyperpolarizes vascular smooth muscle cells and endothelial cells both from wild-type and CSE-knock out mice. In particular, potassium channels seem to also be involved in this mechanism because the authors observed that small-conductance potassium channels SK2.3 expression was increased by H_2_S and decreased in CSE KO or in wild-type mice treated with CSE inhibitors. As a further confirmation of *S*-persulfidation role in H_2_S-evoked vasodilation the authors reported that -SH oxidants and -SSH inhibitor induced a suppression of the H_2_S-induced hyperpolarization [38]. The activation of ion channels also represents the basis for another mechanism accounting for the H_2_S-induced vasodilation. Indeed, it has been demonstrated that, the NO biosynthesized by the endothelial NOS (eNOS) of the meningeal arteries and the H_2_S produced by the CBS in perivascular nerve fibers, react to give the nitroxyl, HNO. HNO activates transient receptor potential ankyrin 1 (TRPA1) channel, evoking a Ca^2+^-induced release of calcitonin gene related peptide (CGRP) which, in turn, activates its receptors and causes arterial vasodilation. This mechanism demonstrated that the interaction between NO and H_2_S can lead to the formation of nitroxyl HNO, increasing meningeal blood flow and suggesting that the HNO-TRPA1-CGRP pathway could play a significant role in the pathophysiology of headache associated with vasodilation [39]. A cross-talk between NO and H_2_S has also been demonstrated on soluble guanylate-cyclase (sGC) redox state. Indeed, sGC is the main target by which NO induces vasodilation and NO mainly binds sGC when the sGC heme moiety is in the ferrous state. In an interesting study, Zhou and colleagues, demonstrated that H_2_S, being a reducing agent, is able to convert the prosthetic heme group of sGC from Fe^3+^ to Fe^2+^, increasing the pool of sGC which could be activated by NO. Therefore, according to this study, H_2_S and H_2_S-donors support the NO-induced vasodilation, by assuring a continuous reduction of ferric sGC heme into a ferrous state [40]. In recent studies, a further mechanism of action, accounting for the vasorelaxing effect of H_2_S, has been described. The activation of VEGFR2 by H_2_S has been investigated as a possible mechanism of action accounting both for the anti-hypertensive effect due to GYY4137 (a slow H_2_S-donor) on spontaneously hypertensive rats (SHR) and the vasodilation exhibited by the H_2_S-donor NaHS in rat cerebral basilar artery (CBA) [17,18]. These studies demonstrated that GYY4137 induced vascular protection and anti-hypertensive effect through upregulating the expression of VEGFR2, which in turn reduces the endothelial dysfunction in SHR. In contrast, in CBA vascular smooth muscle, the vasodilation induced by NaHS was strongly attenuated when the expression of VEGFR2 was knocked down. Moreover, the H_2_S-induced vasorelaxing effect was also decreased when the animals were treated with VEGFR2-blockers, suggesting an involvement of this target in the vasodilating effect induced by H_2_S and H_2_S-donor at CBA level [18] (Figure 1).

## 2. H_2_S Releasing Mechanism of Polysulfides and Isothiocyanates

**Figure 2:**
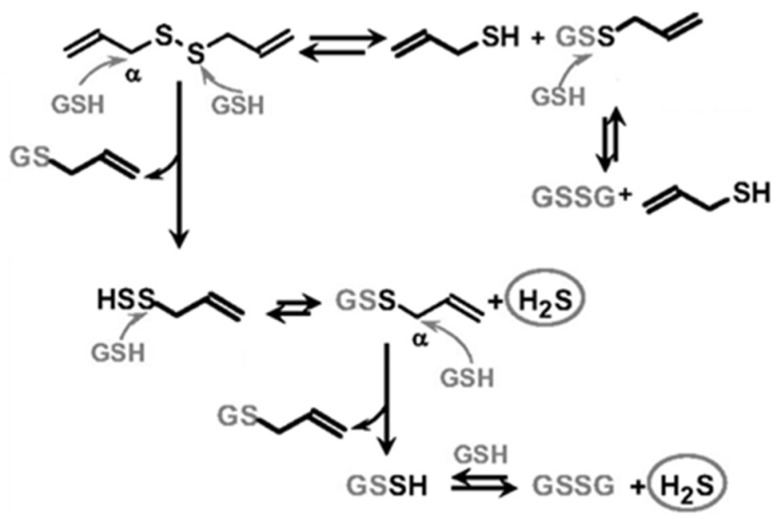
Reaction between diallyl disulfide and glutathione (GSH), yielding the formation of H_2_S and perthiols, which generate another molecule of H_2_S and glutathione disulfide (GSSG).

In the last few years, the interest in developing chemical moieties behaving as “slow” H_2_S-donors has been growing due to the plethora of pharmacological effects exhibited by this gaseous molecule and the impossibility to directly administrate gaseous H_2_S or sulfur salts [21]. Although NaHS, Na_2_S, and CaS salts effectively and rapidly generate H_2_S, they have only been used for experimental purposes since their H_2_S kinetic release does not allow for clinical employment due to potential severe side effects caused by difficulties in in the control of the dosage [41]. The development of novel synthetic H_2_S chemical moieties still represents a fundamental strategy for implementing the pharmacological armamentarium in the treatment of those pathologies characterized by an impaired production of H_2_S, including hypertension [42]. This approach led to the discovery, synthesis and pharmacological investigation of several H_2_S donors characterized by heterogeneous chemical structures (i.e., thiamides, iminothioethers, thioureas, and thiols) [34,43,44,45,46,47,48,49], further elucidating the cardiovascular effect of H_2_S and revealing the importance of a “slow” and, probably, endogenous-like kinetic release. Researchers have also focused their attention on natural sulfur compounds, polysulfides derived from *Alliaceae*—diallyl disulfide (DADS) and diallyl trisulfide (DATS)—and isothiocyanates (ITCs) produced from the myrosinase-dependent metabolism of glucosinolates (GLS) contained in the *Brassicaceae* family, which includes many edible plants, such as broccoli, rocket salad, and cabbage. Notably, all these organosulfur compounds, although structurally heterogeneous, exhibit biological effects that consistently overlap those exerted by H_2_S [50]. In 2007, Benavides and colleagues demonstrated for the first time that the real mediator of the antihypertensive effect related to a garlic-rich diet was the gaseous molecule H_2_S. Garlic has a high content of organic polysulfide compounds, such as DADS and DATS, which behave as H_2_S-releasing compounds in a thiol-dependent manner, mediating the vaso-activity of garlic. DADS and DATS promoted vascular smooth muscle relaxation in phenylephrine (PE)—precontracted aorta rings suspended in buffer solutions containing 1 mM glutathione (GSH). This resulted in concentration-dependent simultaneous vasorelaxation and H_2_S production suggesting a link between bioactivity and production of this signal molecule. Furthermore, the authors demonstrated a chemical reaction between GSH and garlic-derived polysulfides, which cross cell membranes, react with GSH to induce a nucleophilic substitution at the α carbon, leading to the formation of *S*-allyl-glutathione and allyl perthiol, which in turn undergo nucleophilic substitution at the *S*-atom, yielding allyl-glutathione disulfide (GSSG) and H_2_S (Figure 2) [50].

**Figure 3:**
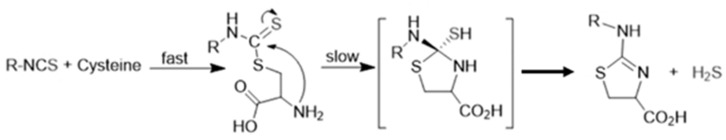
Schematic representation of the reaction between cysteine and organic isothiocyanates (ITCs) leading to the formation of H_2_S.

Among naturally occurring sulfur compounds, ITCs derived by the metabolism of glucosinolates contained in the *Brassicaceae* family recently emerged as an intriguing chemotype of interest in cardiovascular pharmacology research. As demonstrated for DADS and DATS, which can be considered as H_2_S donor prodrugs, ITCs also showed pharmacological effects similar to those of H_2_S [51]. The hypothesis that ITCs may behave as H_2_S-releasing agents came from the close overlap between many physiological/biological effects attributed to ITCs (often shared by many different ITCs, irrespective of their structural differences) and those exhibited by the gasotransmitter H_2_S. Both ITCs and H_2_S behave as antioxidant and anti-inflammatory agents, are activators of potassium channels modulating a vasodilator effect, are well-known chemopreventive agents, etc. Starting from this observation, in 2014 Citi and colleagues amperometrically evaluated H_2_S release by several natural ITCs (allyl isothiocyanate (highly present in black mustard, *Brassica nigra* L.), 4-hydroxybenzyl isothiocyanate (HBITC, highly present in white mustard, *Sinapis alba* L.), benzyl isothiocyanate (BITC, highly present in garden cress, *Lepidium sativum* L.), and erucin (ERU, present in different species such as broccoli, *Brassica oleracea* L., and rocket, *Eruca sativa* Mill.) and observed a slow and thiol-dependent H_2_S release, revealing, as for polysulfide, that the beneficial effect of ITCs was due to gaseous H_2_S [52]. Wang et al. in 2018 reported the H_2_S-releasing properties of moringin, an ITC derived from *Moringa oleifera* Lam., a plant belonging to *Moringaceae* family. The authors analyzed the different content of GLS and ITCs in different Moringa tissues and measured the H_2_S-releasing properties of the extracts with the lead acetate test, by exploiting the high affinity of divalent lead and H_2_S to form a black precipitate (PbS) [53]. The authors reported that Moringa seeds (seeds with shell and seed kernels) rich in 4-*O*-(-l-rhamnopyranosyloxy)-benzylglucosinolate, which is converted into the secondary metabolite isothiocyanate (mainly BITC), effectively released H_2_S in the presence of an excess of l-cysteine [53]. Lucarini and colleagues also reported that the ITC sulforaphane (SFN), derived from the myrosinase-mediated hydrolysis of glucoraphanine, exhibited l-cysteine-dependent H_2_S donation [54]. Better understanding of the molecular reactivity responsible for the cysteine-mediated H_2_S release from isothiocyanates has been provided by Lin and co-workers [55]. They determined that ITCs rapidly form adducts with cysteine. These adducts undergo intramolecular cyclization followed by releasing organic amine R–NH_2_ and raphanusamic acid (RA) as major products with formation of H_2_S and 2-carbylamino-4,5-dihydrothiazole-4-carboxylic acids as minor products (Figure 3).

These preliminary results of the H_2_S-releasing properties of polysulfide compounds and natural ITCs, respectively derived from *Alliaceae*, *Brassicaceae,* and *Moringa*, and the characterization of the molecular mechanism leading to the formation of H_2_S in the presence of free thiols, established the basis for further pharmacological investigations about their potential antihypertensive effects. In the following paragraphs, the antihypertensive properties, starting from preclinical evidence and moving to the clinical effects, are described demonstrating the nutraceutical potential of these compounds.

## 3. Antihypertensive Effects of Garlic and Garlic Polysulfides in Preclinical Studies

The potential antihypertensive effects of garlic and its organosulfur derivatives have been widely demonstrated, and many mechanisms of action (including those mediated by H_2_S) for this edible plant have been proposed. In 2003, Sharifi and colleagues examined the pharmacological effects of garlic in an animal model of hypertension induced by surgery [56]. In this study, two-kidney-one-clip (2K1C) hypertensive rats were treated daily with an aqueous extract of garlic (50 mg/kg/day, orally) for 4 weeks. Blood pressure levels, measured every week by the tail-cuff method, were significantly reduced in the animals that received extract of garlic compared to the control group. Interestingly, the beneficial effects of garlic were clearly visible at one week after beginning of treatment, and they were mainly associated with a reduction in angiotensin I-converting enzyme (ACE) activity in several organs and tissues (i.e., kidney, aorta, heart, and lung). It is noteworthy that H_2_S also exhibited inhibitory effects on ACE expression and activity in endothelial cells [57]. Nwokocha and colleagues used the same experimental model of hypertension to investigate the acute antihypertensive effects of an aqueous garlic extract in both normotensive and 2K1C rats [58]. In this study, the intravenous administration of garlic extract (5–20 mg/kg) led to significant dose-dependent decreases in blood pressure in both the normotensive and 2K1C models. Similar antihypertensive effects have been also demonstrated in other animal models of hypertension. For instance, a single daily dose of processed garlic (30–50 mg/kg) administered for 8 weeks promoted antihypertensive properties in spontaneously hypertensive rats (SHR) [59]. Processed garlic, containing 75.3 mg/100 g of *S*-allylcysteine (SAC), significantly, although dose-independently, prevented the progressive increase in systolic and diastolic blood pressure levels observed in the control group. In another study on SHR, both aged garlic extract and raw garlic significantly reduced blood pressure levels after 10 weeks of treatment (dosage unknown) [60]. Very recently, black garlic extract standardized in the organosulfur compounds DAS (87.8 µg/g), DADS (203.9 µg/g) and DATS (282.6 µg/g) exhibited antihypertensive effects in a rat model of deoxycorticosterone acetate salt-induced hypertension [61]. Two dosages of black garlic extract (50 and 100 mg/kg) were administered to hypertensive animals once a day for 7 weeks by tube feeding, and compared with the antihypertensive drug lisinopril as reference compound. Results showed that both dosages of garlic extract markedly reduced systolic blood pressure in a dose-dependent manner. As previously described, the crucial role of H_2_S in the vasoactive effects of garlic and its derivatives was observed for the first time by Benavides and colleagues in 2007, who demonstrated that both human red blood cells and intact rat aorta rings are able to convert garlic-derived polysulfides into H_2_S [50]. Moreover, they observed that the vasorelaxing effects promoted by garlic and DADS (100 µM) on isolated rat aortic rings were dependent on the concentrations of H_2_S released. More recently, Hsu and colleagues also proposed a potential role for H_2_S in the antihypertensive properties of garlic [62]. They demonstrated that maternal garlic oil supplementation (100 mg/kg/day) during pregnancy and lactation significantly prevents high-fat diet-induced hypertension in 16-week-old male rat offspring. In these experimental conditions, the high-fat diet led to a reduction in the activity of H_2_S-generating enzymes in the kidney, with a consequent marked decrease in plasma H_2_S levels. Maternal garlic oil supplementation significantly prevented this fall in plasma H_2_S levels by increasing renal mRNA expression and activity of enzymes involved in the endogenous production of H_2_S. Moreover, garlic oil increased nitric oxide (NO) bioavailability and altered gut microbiota composition. These results, although preliminary, reveal a possible association between the H_2_S-generating pathway in the kidneys, NO system and gut microbiota in hypertension induced by a high-fat diet, as well as a potential modulatory effect on the endogenous “H_2_S system” by garlic. Besides modulation of the NO pathway, Ashraf and colleagues proposed an additional mechanism of action for garlic that involves KATP channels [63]. Pre-treatment of rat aortic rings with the KATP channel blocker glybenclamide significantly attenuated the vasorelaxing properties exhibited by garlic (1–50 µg/mL), suggesting the involvement of KATP channels in the vasoactive effects. Intriguingly, the same mechanism of action has been widely described for both H_2_S and H_2_S-donors [14].

The possible involvement of H_2_S in the antihypertensive properties of garlic has also been suggested for the H_2_S-donor DATS [64] which exhibited antihypertensive effects in Wistar rats with metabolic syndrome probably through modulation of the “H_2_S system” [65]. In fact, daily administration of DATS (40 mg/kg/day for 3 weeks, orally) led to a decrease in systolic blood pressure and significantly enhanced serum H_2_S levels, which were dramatically reduced in rats with metabolic syndrome compared with healthy controls. In addition, DATS prevented the development of hyperhomocysteinemia in rats with metabolic syndrome, a pathological condition that markedly influences H_2_S metabolism and predisposes to hypertension [66]. Recently, a potential H_2_S-mediated mechanism of action has also been described for allicin, a garlic-derived organosulfur compound with low stability in aqueous media that rapidly decomposes into four H_2_S-releasing compounds, DAS, DADS, DATS, and ajoene [41]. Allicin (2.50–15.77 mM) has been reported to produce a concentration-dependent vasorelaxation on rat mesenteric arterial rings that was reduced by pre-incubation with the CSE inhibitor L-propargylglycine (PAG) [67]. The vasorelaxing effects of allicin also involved the modulation of the “NO-system”, as cyclic guanosine monophosphate (cGMP) and cyclic adenosine monophosphate (cAMP) levels increased after incubation of mesenteric arterial rings with allicin. Once again, pre-incubation of PAG significantly reduced these effects and the removal of endothelium led to a decline in allicin-induced vasorelaxation. Interestingly, a possible crosstalk between NO and H_2_S in the cardiovascular system has been reported [10,68]. Finally, in in vivo experiments, the antihypertensive effects exhibited by allicin in SHR (7–14 mg/kg/day for 4 weeks) were significantly attenuated in the presence of PAG [67]. The antihypertensive properties of allicin have also been demonstrated in other preclinical studies, independent from the “H_2_S hypothesis”. For instance, daily treatment with allicin significantly reduced systolic blood pressure in dexamethasone-induced hypertensive rats (8 mg/kg/day for 8 weeks, oral allicin) [69], in a rat model of hypertension induced by high-fructose diet (8 mg/kg/day for 2 weeks, oral allicin) [70] and in hypertensive rats with chronic kidney disease (40 mg/kg/day for 6 weeks, oral allicin) [71]. In conclusion, all these studies suggest a possible link between garlic and H_2_S, due to the antihypertensive effects promoted by H_2_S, garlic, and its H_2_S-donor derivatives being highly superimposable and the involvement of the modulation of common H_2_S-related signaling pathways (Table 1).

## 4. Antihypertensive Effects of Isothiocyanates in Preclinical Studies

Although interest in investigating the potential pharmacological effects of ITCs grew with the discovery of their ability to release H_2_S, the vasoactive properties of natural ITCs had previously been reported [72]. The vasorelaxing effect of ITCs was first evaluated using BITC (the active compound of papaya seed extract). BITC promoted vasorelaxing effects on tissue strips pre-contracted with phenylephrine (PE) and limited KCl- or PE-induced contractions, with no correlation to the simultaneous release of H_2_S [73]. *Eruca sativa* Mill., a widely studied cruciferous vegetable rich in GLS, and thus able to furnish a high amount of ITCs, has been reported to decrease arterial pressure in rats [74]. Intravenous injection of crude extract decreased arterial pressure values in both normotensive (maximum decrease: 41.79 ± 1.55% mmHg) and SHRs (maximum decrease: 58.25 ± 0.91% mmHg), an effect that was significantly attenuated by atropine (1 mg/kg) pretreatment supporting the involvement of muscarinic receptors in the antihypertensive effect. In rat isolated aortic rings from normotensive rats, crude extract of Eruca induced endothelium-dependent relaxation that was partially inhibited by treatment with *N*-nitro-arginine methyl ester (l-NAME), atropine or after endothelium removal, revealing the important role of muscarinic receptor-linked NO production. In aorta from the SHRs, crude extract induced endothelium-independent relaxation that was not affected by pretreatment with l-NAME or atropine. Phytochemical analysis revealed the presence of phenols and flavonoids, whereas HPLC analysis of crude extract indicated the presence of the isothiocyanate erucin [74]. The vasorelaxing effects and thus the antihypertensive properties of natural ITCs related to their ability to release H_2_S was firstly reported by Martelli et al. who described the vascular effects of erucin, the ITC derived from *Eruca sativa* Mill. [72,75]. In this work the authors demonstrated that erucin released H_2_S into HASMCs in a concentration-dependent manner, clarifying the mechanism of action responsible for the widely reported antihypertensive effect of *Eruca sativa* Mill. Indeed, erucin induced a clear hyperpolarizing effect in HASMCs, a significant vasorelaxing effect in endothelium-denuded vessels which was greater in endothelium-intact rat aortic rings and the inhibition of norepinephrine-induced contraction, showing important vasoactive properties. As a final demonstration of its antihypertensive effects, erucin (10 mg/kg) promoted a significant reduction of blood pressure in SHRs, restoring blood pressure to values similar to those observed in normotensive rats. A slight and non-significant lowering of systolic blood pressure was observed in normotensive rats [72]. A recent study reported the antihypertensive effect of *Semen Brassicae* (i.e., the *Sinapis alba* L. or *Brassica alba* L. semen, the seeds from which mustard is obtained) treatment [76]. *Semen Brassicae* gavage treatment (0.5 g/kg, 1.0 g/kg or 2.0 g/kg water-decocted solution from *Semen Brassicae* diluted in distilled water (10 mL/kg) once a day for 8 weeks) significantly decreased blood pressure in SHRs, and also evoked a significant reduction in oxidative stress, a marked inhibition in endothelin-1 production and a limited inflammatory response [76]. *Moringa oleifera* leaf extract (MOE) has also been reported to reduce blood pressure. MOE contains high levels of GLS and ITCs and has in vitro antioxidant capacity. The researchers reported that treatment with MOE (30 and 60 mg/kg/day) decreased arterial blood pressure in a dose-dependent manner. MOE decreased the impairment of acetylcholine-induced relaxation and reduced adrenergic-induced contraction in isolated mesenteric arterial vessels. In addition, MOE (0.001–0.3 mg) produced a concentration-dependent relaxation in methoxamine pre-contracted isolated aortic rings from SHRs [77] (Table 2).

## 5. Antihypertensive Effects of Garlic in Humans

The potential antihypertensive effects of garlic have been demonstrated in many clinical studies. A recent meta-analysis of 12 randomized clinical trials [78] reported a mean ± standard error decrease of 8.3 ± 1.9 mmHg in systolic blood pressure and 5.5 ± 1.9 mmHg in diastolic blood pressure in hypertensive subjects treated with garlic. On the contrary, a previous meta-analysis by the same author showed that garlic treatment does not change blood pressure levels in pre-hypertensive or normotensive subjects [79]. One-third of participants generally reported mild side effects associated to chronic therapy with garlic supplements, which include reflux, flatulence, and burping [80,81]. More severe gastrointestinal effects with therapeutic dosages of garlic were reported by a small percentage of patients [80,81,82]. These data indicate that garlic supplements can be considered as a safe option for the management of cardiovascular diseases, including hypertension. After publication of Ried’s systematic review in 2020 [78], two other clinical studies on the antihypertensive effects of garlic in humans have been published. Kravchuk and colleagues conducted a clinical trial on 10 middle-aged hypertensive men who received garlic supplement (400 mg/day for 30 days) after an initial treatment period with standard antihypertensive therapy with β-blockers or ACE inhibitors [83]. At the end of the treatment, garlic reduced both systolic and diastolic blood pressure by 16.5 and 12.5 mmHg from the baseline, respectively. Interestingly, blood levels of H_2_S at the baseline were about 50% lower in hypertensive subjects compared with healthy individuals. The initial treatment with antihypertensive drugs led to a further reduction in endogenous H_2_S production, which was restored in the hypertensive patients who consumed garlic daily for 30 days in combination with standard antihypertensive therapy. Noteworthy, garlic also showed promising cholesterol-lowering and mild antithrombotic properties in participants with hypertension, thus acting as a potential multi-target agent in the clinical management of hypertension and concomitant cardiovascular risk factors. In this regard, a previous clinical trial demonstrated that garlic supplements are also effective in reversing the aging of the arteries, thus preventing arterial stiffness in hypertensive subjects. This latter effect further contributes to the potential cardiovascular protective properties of garlic and its derivatives [84]. Soleimani and colleagues investigated the effects of garlic supplements on blood pressure in patients with non-alcoholic fatty liver disease (NAFLD) in a randomized, double-blind, placebo-controlled clinical trial [85]. The study was completed by 47 patients allocated to the garlic group (400 mg garlic tablet containing 1.5 mg allicin, twice daily for 15 weeks) and 51 patients in the placebo group. Importantly, some patients in both the intervention and placebo arms were not hypertensive, and the results reported by the authors do not distinguish the clinical effects of garlic in these two groups of patients. Daily treatment with garlic led to a mean reduction of 6.8 mmHg in systolic blood pressure and 5.0 mmHg in diastolic blood pressure from the baseline. Conversely, there was no significant change in blood pressure values observed in the placebo group. Finally, in a randomized, placebo-controlled clinical trial not included in the meta-analysis by Ried [78], daily consumption of processed garlic (1 g daily for 8 weeks, corresponding to 0.8 mg of SAC/day) significantly reduced systolic blood pressure in 23 hypertensive subjects (about 8 mmHg) from the baseline [59]. The antihypertensive effects of garlic were observed after 2 weeks of treatment. Once again, there were no significant effects in the placebo group. Similarly, Ashraf and colleagues demonstrated that garlic tablets (300–1500 mg/day for 24 weeks) were effective in reducing both systolic and diastolic blood pressure in 150 patients with essential hypertension in a dose-dependent manner (about 8 mmHg in patients treated with the highest dosage of garlic) [86]. Interestingly, the reduction in systolic blood pressure was quantitively superimposable to that promoted by the reference antihypertensive drug atenolol (50–100 mg/day). These results strongly indicate that garlic is a safe and effective option for the clinical management of hypertension. However, both garlic supplement type and dosage of the active ingredient employed in these clinical studies were very heterogeneous, thus limiting a clear and uniform interpretation of the observed effects. Most trials used standard garlic powder supplements, aged garlic extract or garlic oil for a highly variable treatment period (from 2 to 24 weeks) at different dosages, corresponding to 7.8–11.7 mg/day for alliin, 3.0–31.2 mg/day for allicin or 0.8–2.4 mg/day for SAC [59,79,85]. Therefore, given the heterogeneous experimental conditions employed, further clinical studies are needed to confirm this promising clinical evidence (Table 3).

## 6. Antihypertensive Effects of Broccoli in Humans

The potential antihypertensive properties of *Brassicaceae* edible plants in humans are still nebulous and the results of the few clinical studies available are quite conflicting. In fact, the published trials show very heterogeneous experimental conditions, from selected population/intervention to primary clinical outcome considered. A randomized clinical trial by Christiansen and colleagues [96] showed that treatment of 20 hypertensive patients with 10 g/day dried broccoli sprouts (equivalent to 100 g fresh sprouts) for 4 weeks led to a small but non-significant decrease in systolic blood pressure (about 8 mmHg from the baseline). No change in diastolic blood pressure has been observed in this group of patients. In a recent study, 12 women with pregnancy hypertension received a myrosinase-activated broccoli seed extract (BroccoMax^®^), which is equivalent to 32 mg of sulforaphane [97]. The protocol involved six pregnant women taking four BroccoMax^®^ capsules and six women taking eight capsules, without interrupting their standard antihypertensive medication of nifedipine or labetalol. Systolic and diastolic blood pressure were recorded for 8 h after ingesting the BroccoMax^®^ capsules, but no significant acute effects of broccoli have been observed on systolic blood pressure in pregnant women with hypertension. However, results showed a modest (about 10%) reduction in diastolic blood pressure over time, especially after ingestion of the highest dose of BroccoMax^®^. Finally, in a randomized clinical trial involving 86 type 2 diabetic patients with hypertension and a diagnosis of *Helicobacter pylori* infection, daily consumption of broccoli sprouts powder (BSP) in addition to standard triple therapy (STT) for *H. pylori* eradication was associated with promising antihypertensive effects [98]. Patients were treated with STT (omeprazole 20 mg, clarithromycin 500 mg, amoxicillin 1000 mg) twice a day for 14 days, BSP (6 g/day) for 28 days or combination of STT and BSP for 4 weeks. Twenty-five patients belonging to the BSP group and 24 patients of the STT + BSP group completed the study. At the end of treatment, both systolic and diastolic blood pressure were significantly decreased in patients treated with STT plus BSP, but not with either BSP or STT alone. These interesting results suggest that BSP might effectively reduce blood pressure levels in hypertensive patients, but it is difficult to explain why this effect was not clear in patients treated with BSP alone. Probably, the participants of this clinical trial do not reflect the hypertensive population, as only hypertensive patients with concomitant type 2 diabetes and *H. pylori* infection were recruited for this study. Therefore, these multiple pathological conditions may have masked the effects of treatment with BSP alone. Interestingly, three recent prospective cohort studies of about 190,000 subjects with more than 20 years of follow-up investigated the association between individual fruits and vegetables consumption and incidence of hypertension [99]. Results showed that regular consumption (½ cup for more than four times per week) of broccoli, but not cauliflower or Brussels sprouts, was associated with significant reduced risk of incident hypertension. Conversely, there was no association between a low consumption of these vegetables (less than one serving per month) and reduced risk of hypertension. Importantly, this study considered both raw and cooked vegetables, without distinguishing between methods of cooking. Evidently, this aspect might affect the biological effects of edible plants, as processing and cooking markedly reduce the bioavailability of many metabolites, including isothiocyanates from *Brassicaceae* [100,101]. Another limitation of this study was the diagnosis of hypertension, which was self-reported and not performed by a physician. Taken together, this very heterogeneous clinical evidence does not allow delineation of a precise pharmacological profile for *Brassicaceae* in this field. Hence, further studies are strongly encouraged to better elucidate the potential role of these edible plants in both prevention and clinical management of hypertension (Table 4).

## 7. Conclusions

H_2_S is the most-recent gasotransmitter to be discovered after NO and CO. Starting from its description as endogenous gaseous modulator by Abe and Kimura in the 1996, many researchers focused their attention on the characterization on the physiological role, the mechanisms of action and potential exogenous sources, i.e., H_2_S-donors, able to make up for the lack or the imbalance of H_2_S observed in some pathological conditions. Despite its first recognition in the central nervous system, the cardiovascular system is a field that has attracted attention with regards to H_2_S, probably because of similarities with the cardiovascular profile of the most famous gasotransmitter, NO. This interest on the cardiovascular effect of H_2_S led to clarification that, although the final effects of H_2_S and NO on heart and vessels are similar, the mechanisms of action and the ancillary properties are very different. Indeed, H_2_S has powerful antioxidant properties and modulates several proteins (i.e., potassium channels and enzymes) through the *S*-persulfidation process. The impact of H_2_S potency on vascular tone is lower than that exerted by NO. However, its lower, slow, long-lasting effects could be more effective in the treatment of chronic diseases such as hypertension than that exhibited by NO which is mainly used in the treatment of angina’s acute vasospasm. In this scenario, the research of exogenous sources of H_2_S became a challenge in cardiovascular pharmacology and some interesting findings arrived through the natural, edible, sources of H_2_S, the so-called sulfur nutraceuticals. Among them, the *Alliaceae* (e.g., garlic and onions) were firstly known as anti-hypertensive edible plants and, after the discovery of H_2_S, their vasoactive mechanism of action has been revealed as vasodilation due to the H_2_S-donor properties exhibited by, for example, garlic polysulfides. *Brassicaceae* or Crucifers are mainly known as anti-cancer agents and their effect as anti-hypertensive agents were demonstrated only recently, almost contemporaneously with the discovery that isothiocyanates deriving form *Brassicaceae* are real H_2_S-donor moieties. Currently, many preclinical studies support the anti-hypertensive effects of *Alliaceae* or *Brassicaceae* (or of their active principles, i.e., polysulfides or isothiocyanates) and several of these studies confirmed that this vascular effect was due to their ability to release H_2_S. However, as concerns the clinical evidence, the use of natural H_2_S-donors in the management of hypertension or pre-hypertension is more supported for garlic while is still controversial for *Brassicaceae* or Crucifers. The need to further elucidate the potential use of *Brassicaceae* as natural H_2_S-donors in the management of hypertension derives from the availability of few heterogeneous studies in which the presence of comorbidities or polytherapy did not allow a clear comprehension of the real impact of these edible plants. Moreover, many *Brassicaceae* need to be cooked before eating and this process leads to myrosinase denaturation with a consequent lowering of isothiocyanates availability. Hence, future studies should also consider this factor or bypass it using standardized supplements. In conclusion, the potential use of natural H_2_S-donors in the management of hypertensive diseases seems to have a solid pharmacological basis even if further studies are necessary to elucidate their real impact on the prevention and on the clinical management of hypertension.

## Figures and Tables

**Table 1:** Antihypertensive effects of garlic and garlic polysulfides in preclinical studies. Blood pressure (BP) values at the end of treatment were reported for studies evaluating the preventive effects of supplements against BP increase in hypertensive animals, while change (%) in BP was reported for studies directly evaluating the blood pressure-lowering effects of garlic or garlic polysulfides in animals with hypertension. All values refer to systolic BP (mmHg) reported as mean ± SEM.

First Author, Year	Experimental Model	Treatment	Daily Dose (mg/kg)	Time	BP in the Control Group at the End of Treatment	BP in the Treated Group at the End of Treatment	BP Change (%) from the Baseline
Chen, 2021 [61]	Deoxycorticosterone acetate salt-induced hypertensive rats	Black garlic extract	50, orally	7 weeks	173.4 ± 1.8	155.0 ± 3.2	-
Chen, 2021 [61]	Deoxycorticosterone acetate salt-induced hypertensive rats	Black garlic extract	100, orally	7 weeks	173.4 ± 1.8	150.0 ± 3.0	-
Cui, 2020 [67]	Spontaneously hypertensive rats	Allicin	7, orally	4 weeks	194.20 ± 8.6	168.22 ± 2.6	-
Cui, 2020 [67]	Spontaneously hypertensive rats	Allicin	14, orally	4 weeks	194.20 ± 8.6	141.01 ± 2.5	-
Dubey, 2017 [69]	Dexamethasone-induced hypertensive rats	Allicin	8, orally	8 weeks	133.6 ± 0.8	103.8 ± 1.9	-
Elkayam, 2001 [70]	High-fructose diet-induced hypertensive rats	Allicin	8, orally	2 weeks	152.4 ± 3.9	139.7 ± 12.0	−8.9 ± 7.8
Garcia-Trejo, 2016 [71]	Hypertensive rats with chronic kidney disease	Allicin	40, orally	6 weeks	Significant antihypertensive effects *		
Han, 2011 [59]	Spontaneously hypertensive rats	Processed garlic	30–50, orally	8 weeks	Significant antihypertensive effects *		
Harauma, 2006 [60]	Spontaneously hypertensive rats	Aged garlic extract/raw garlic	Unknown	10 weeks	Significant antihypertensive effects *		
Hsu, 2021 [62]	High-fat diet-induced hypertensive rats	Garlic oil	100, orally (maternal supplementation)	During pregnancy and lactation	153.0 ± 1.0	139.0 ± 1.0	-
Jeremic, 2020 [65]	High-fat diet-induced hypertensive rats	Diallyl trisulfide	40, orally	3 weeks	Significant antihypertensive effects *		
Nwokocha, 2011 [58]	Two-kidney-one-clip hypertensive rats	Garlic extract	20, intravenously	Acute administration (30 min)	-	-	16.7 ± 2.0
Sharifi, 2003 [56]	Two-kidney-one-clip hypertensive rats	Garlic extract	50, orally	4 weeks	Significant antihypertensive effects *		

* In the original article, results were statistically significant but were shown only in the graphical form and not as mean ± SEM.

**Table 2:** Antihypertensive effects of *Brassicaceae*, *Moringaceae,* and isothiocyanates (erucin) in preclinical studies. Blood pressure (BP) values at the end of treatment were reported for studies evaluating the preventive effects of supplements against BP increase in hypertensive animals, while change (%) in BP was reported for studies directly evaluating the blood pressure-lowering effects of supplements in animals with hypertension. All values refer to systolic BP (mmHg) reported as mean ± SEM. WD: water decocted.

First Author, Year	Experimental Model	Treatment	Daily Dose	Time	BP in the Control Group at the End of Treatment	BP in the Treated Group at the End of Treatment	BP Change (%) from the Baseline
Aekthammarat, 2019 [77]	L-NAME-induced hypertensive rats	*Moringa oleifera* leaf extract	30 mg/kg, orally	3 weeks	189.9 ± 2.1	177.0 ± 2.7	-
Aekthammarat, 2019 [77]	L-NAME-induced hypertensive rats	*Moringa oleifera* leaf extract	60 mg/kg, orally	3 weeks	189.9 ± 2.1	152.0 ± 0.7	-
Lin, 2020 [76]	Spontaneously hypertensive rats	WD solution from *Semen Brassicae*	0.5 g/kg, orally	8 weeks	192.2 ± 2.6	128.7 ± 2.3	-
Lin, 2020 [76]	Spontaneously hypertensive rats	WD solution from *Semen Brassicae*	1 g/kg, orally	8 weeks	192.2 ± 2.6	118.7 ± 2.6	-
Lin, 2020 [76]	Spontaneously hypertensive rats	WD solution from *Semen Brassicae*	1 g/kg, orally	8 weeks	192.2 ± 2.6	104.6 ± 1.8	-
Martelli, 2020 [72]	Spontaneously hypertensive rats	Erucin	10 mg/kg, intraperitoneally	Acute administration (2 h)	-	-	−23.9 ± 3.8
Salma, 2018 [74]	High salt (NaCl)-induced hypertensive rats	Crude extract of *Eruca sativa* Mill.	1 mg/kg, intravenously	Acute administration	-	-	−25.4 ± 3.9
Salma, 2018 [74]	High salt (NaCl)-induced hypertensive rats	Crude extract of *Eruca sativa* Mill.	3 mg/kg, intravenously	Acute administration	-	-	−39.2 ± 1.8
Salma, 2018 [74]	High salt (NaCl)-induced hypertensive rats	Crude extract of *Eruca sativa* Mill.	10 mg/kg, intravenously	Acute administration	-	-	−46.8 ± 3.6
Salma, 2018 [74]	High salt (NaCl)-induced hypertensive rats	Crude extract of *Eruca sativa* Mill.	30 mg/kg, intravenously	Acute administration	-	-	−58.3 ± 0.9
Salma, 2018 [74]	High salt (NaCl)-induced hypertensive rats	Crude extract of *Eruca sativa* Mill.	30 mg/kg, orally	Acute administration	-	-	−40.3 ± 1.2
Salma, 2018 [74]	High salt (NaCl)-induced hypertensive rats	Crude extract of *Eruca sativa* Mill.	100 mg/kg orally	Acute administration	-	-	−59.4 ± 0.8

**Table 3:** Antihypertensive effects of garlic and garlic polysulfides in clinical studies. All values refer to systolic blood pressure (BP) expressed in mmHg and reported as mean ± SEM.

First Author, Year	No. of Subjects in the Experimental Group	Treatment	Daily Dose (mg)	Time (Weeks)	Change in BP from the Baseline
Ashraf, 2013 [86]	30	Garlic tablets	300	24	−2.3 ± 0.9
Ashraf, 2013 [86]	30	Garlic tablets	600	24	−4.3 ± 1.0
Ashraf, 2013 [86]	30	Garlic tablets	900	24	−6.1 ± 1.0
Ashraf, 2013 [86]	30	Garlic tablets	1200	24	−6.7 ± 1.2
Ashraf, 2013 [86]	30	Garlic tablets	1500	24	−7.6 ± 0.9
Auer, 1990 * [87]	20	Garlic powder	600	12	−19.0 ± 3.5
Han, 2011 [59]	23	Processed garlic	500	8	−8.1 ± 2.9
Holzgartner, 1992 * [88]	47	Garlic powder	900	12	−8.0 ± 1.7
Kandziora, 1988 * [89]	20	Garlic powder	600	12	−16.0 ± 1.7
Kravchuk, 2021 [83]	10	Garlic powder	400	4	−16.5 ± 2.6
Nakasone, 2013 * [90]	23	Garlic powder	188	12	−6.6 ± 1.8
Ried, 2010 * [80]	6	Aged garlic extract	960	12	−15.2 ± 2.6
Ried, 2013 * [81]	20	Aged garlic extract	480	12	−2.5 ± 3.7
Ried, 2016 * [91]	38	Aged garlic extract	1200	12	−10.0 ± 1.8
Ried, 2018 * [84]	23	Aged garlic extract	1200	12	−14.3 ± 2.9
De Santos, 1993 * [92]	27	Garlic powder	900	24	−25.0 ± 4.2
Sobenin, 2008 * [93]	23	Garlic powder	600	12	−6.6 ± 1.4
Sobenin, 2009 * [94]	18	Garlic powder	2400	8	−9.3 ± 0.7
Soleimani, 2021 [85]	47	Garlic powder	800	15	−6.7 ± 1.3
Vorberg, 1990 * [95]	20	Garlic powder	900	16	−6.0 ± 2.4

* Included in the systematic review and meta-analysis of randomized clinical trials by Ried, 2020 [78].

**Table 4:** Antihypertensive effects of broccoli in clinical studies. All values refer to systolic blood pressure (BP) expressed in mmHg and reported as mean [95% CI] or mean ± SEM. STT: standard triple therapy (omeprazole 20 mg, clarithromycin 500 mg, amoxicillin 1000 mg) for *H. pylori* eradication.

First Author, Year	No. of Subjects in the Experimental Group	Treatment	Daily Dose (g)	Time	Change in BP from the Baseline
Christiansen, 2010 [96]	20	Dried broccoli sprouts	10	4 weeks	−7.8 [−19.13; 3.53]
Langston-Cox, 2021 [97]	12	Myrosinase-activated broccoli seed extract (BroccoMax^®^)	1–2	8 h	Antihypertensive effects not observed
Mirmiran, 2014 [98]	14	Broccoli sprouts powder	6	4 weeks	−6.0 ± 8.3
Mirmiran, 2014 [98]	22	Broccoli sprouts powder + STT	6	4 weeks	−14.0 ± 5.7

## Data Availability

Not applicable.
